# Public health data quality and evidence use in developing countries: a call to action

**DOI:** 10.3389/fpubh.2023.1194499

**Published:** 2023-07-06

**Authors:** Olan Naz, Muhammad Ibrahim, Amal Fatima Mohiuddin, Adnan Ahmad Khan, Zainab Samad

**Affiliations:** ^1^Research and Development Solutions, Islamabad, Pakistan; ^2^Data, Evaluations and Evidence for Policy (DEEP) Unit, Ministry of National Health Services, Regulations and Coordination, Islamabad, Pakistan; ^3^CITRIC Health Data Science Center, Aga Khan University, Karachi, Pakistan

**Keywords:** health policy, health information system, data quality (DQ), national survey data, program database, evidence use in policymaking

## Introduction

The COVID-19 pandemic highlighted the importance of evidence-based decision-making. However, evidence-guided policy is not the norm in many low and middle-income countries (LMICs), either because the data is not available, or considered unreliable by policymakers who may also not be able to interpret them. Policies created without evidence lead to ineffective programs, wasted resources, and persistently poor health outcomes. Non-availability of high-quality and reliable survey or program data stems both from low capacity and resources to collect and manage data. Irrespective of the reasons, poor quality of data lowers the trust in data among decision makers, who then turn to personal choices and other means (read biases) to make decisions (1–3).

Quality of the data may be poor due to endogenous or institutional causes, such as poor systems or personnel capacity to collect, collate, manage, and process (i.e., analyze and use the results of analysis) accurate data. On the other hand, there may be exogenous constraints such as cultural barriers, lack of interest or awareness of data collection methods, or from a political economy that detracts from arriving at an accurate depiction of the situation on the ground (4). While the latter is a key “logjam” point in evidence use (5), we focus on endogenous constraints as critical entry points to evidence use. Both community surveys and program data that must ideally complement each other are discussed.

Surveys collect information from communities about the health status of populations, and the impact of social and other factors (including public health programs). In Pakistan, as in many other LMICs, several surveys inform about disease priorities that are identified by funding agencies and the government. These include the five-yearly Demographic and Health Survey (DHS), the biennial Pakistan Social and Living Standards Measurement (PSLM) survey and the sporadic Multiple Indicator Cluster Surveys (MICS) that inform against key health indicators at the national or provincial (DHS, PSLM) and district (MICS) levels. Other related datasets include the census that allows the placing of specific populations, including subgroups, on geographic maps, and individual research studies that may address specific questions.

## Survey data

In surveys, while some disease priorities have been consistently replicated over time, key gaps remain. For example, almost all of surveys emphasize communicable diseases and reproductive health, while non-communicable diseases such as hypertension, diabetes, cardiovascular disease, and cancer are noticeably underrepresented. Sometimes priorities change, and questions and modules are dropped or changed in serial surveys, making it difficult to follow certain indicators over time. For example, the module on reasons for non-use of family planning has been changed between two subsequent rounds of DHS in Pakistan, going from reasons for not intending to use (2006–07), to reasons for discontinuation (2012–13 and 2017–18) (6–8). Similarly, information may be incomplete or missing from different surveys. For example, clear definitions of indicators are often not represented in the tools and in some instances relevant variables get omitted.

Data collection through survey questionnaires is always potentially subject to bias (sampling, social desirability, non-response, and recall, including reliance on self-reported behaviors or preferences), illogical variables, monitoring of data collection for quality, and human errors, such as poorly trained enumerators that miss the crux of the question being asked, or simply ignore some questions completely. Sampling issues include strategic omission of certain populations such as men, or worse, systematically passing over subgroups such as working men that are absent during the daytime (9). Other sampling issues include missing out of locations due to expediency or oversight. For example, the question of cost of contraceptives are consistently left unanswered in the DHS. Finally, how surveys are conducted, can account for seemingly similar surveys such as DHS and PSLM, that use the same sampling frame and similar sample size and questions, sometimes yielding highly discrepant indicators. For example, in 2007–08 the fully immunized child percentage was 47% in the DHS and 77% in the PSLM (10).

## Program data

Program data informs the supply side of service delivery (public/private facilities). Typically, government health facility services are tracked in the District Health Information Software (DHIS), while supplies are tracked in the Contraceptive Logistics Management Information System (cLMIS) and Vaccine Logistics Management Information System (vLMIS). In addition, in Pakistan, and possibly in many other countries, private sector provides 75% or more of clinical services, with a substantial portion of services being delivered by large hospital and laboratory chains that can potentially be included in the data net.

Program data fair worse on quality. Such data are often collected by busy people who consider it a secondary responsibility to their primary tasks. Not surprisingly then, data are under or misreported, and one sees data missing for entire districts for some reporting periods. Such issues are worsened by the lack of oversight on program data quality at the site of entries or any feedback from more upstream users. Part of the lack of feedback comes from the fact that much of the reporting is done manually. For example, DHIS initial inputs are through paper-based forms filled once a month, relying on providers' recall of the different types of cases seen in the previous month. Even when such data have been entered, there is seldom feedback on outliers to provide corrections. More importantly, as in surveys, various sources of program data do not match. There are huge discrepancies (sometimes over 10-fold) between DHIS record of clients served and the commodities given during those services; or even between the two systems of contraceptive supply tracking that supposedly receive the same data from the same venues. Eventually, perhaps due to frustration with data issues, different provinces have resorted to their own systems. This causes further confusion as discrepancies, sometimes even definitions, are magnified.

## Triangulation of datasets

A unique problem is consistency in measurement of outcomes between program vs. survey data. For example, in the cases of family planning and childhood immunization, huge differences exist between the magnitude of services from the program data and their supposed uptake as measured in community surveys. For example, the number of intrauterine contraceptive devices given out in 2017–18 was 300% more than the women who said they had received the device in the period in DHS 2017–18, when triangulating the proportion of self-described users against population census. Similar, albeit smaller, differences were present for all types of contraceptives.

## Conclusion

In Pakistan, and possibly in other LMICs, use of evidence in health decisions and confidence of policy and decision makers can be boosted by establishing an “evidence use ecosystem.” The evidence use ecosystem is a comprehensive framework that involves key stakeholders in the production and utilization of evidence to improve health outcomes in a systematic and integrated way (11). The system would receive data from the public and private services and laboratory outlets. It would have dedicated personnel in government, academia or think tanks, who routinely test data for quality through basic error checks and then triangulate different sources of data—to develop and track the overall picture of health and measure the performance of programs that promote it. There should be open and non-judgmental discourse about data quality at acquisition, transmission, and storage stages, as well as for the various analyses and reporting. Policy makers would be facilitated by professionals to understand the data, identify gaps, problems, their solutions, and ways to measure such solutions. These would include innovations such as alternatives to surveys to measure community outcomes, multi-database triangulation, and the use of artificial intelligence applications to predict, track, and control errors, and to make predictions such as forecasting of personnel, supplies, and even infrastructure needs. Ideally, such a system would be decentralized to allow compensation of periodic weaknesses of one or the other component and to avoid monopolization of information by a few actors.

## Author contributions

AK contributed to the conceptualization of the study, acquired funding, and provided critical input for editing and validation of the manuscript. ZS provided valuable input in editing and validation. ON designed and wrote the initial draft of the manuscript, and provided substantial input to editing and refining. MI and AM provided valuable feedback, supervision, and contributed to the editing of the manuscript. All authors have made significant contributions to this piece and have approved the final submitted version.
